# Comparative metabolome analysis of serum changes in sheep under overgrazing or light grazing conditions

**DOI:** 10.1186/s12917-019-2218-9

**Published:** 2019-12-26

**Authors:** Jize Zhang, Yang Gao, Huiqin Guo, Yong Ding, Weibo Ren

**Affiliations:** 1 Key Laboratory of Forage Grass, Ministry of Agriculture, Institute of Grassland Research, Chinese Academy of Agricultural Sciences, Hohhot, 010010 Inner Mongolia China; 2 College of Animal Science and Technology, Jilin Agricultural University, Changchun, Jilin, 130018 China; 3 College of Life Sciences, Inner Mongolia Agricultural University, Hohhot, 010019 Inner Mongolia China; 4 School of Ecology and Environment, Inner Mongolia University, Hohhot, 010021 Inner Mongolia China

**Keywords:** Overgrazing, Light grazing, Serum metabolome, Sheep

## Abstract

**Background:**

Overgrazing is a primary contributor to severe reduction in forage quality and production in Inner Mongolia, leading to extensive ecosystem degradation, sheep health impairment and growth performance reduction. Further studies to identify serum biomarkers that reflect changes in sheep health and nutritional status following overgrazing would be beneficial. We hereby hypothesize that reduced sheep growth performance under overgrazing conditions would be associated with metabolic and immune response alterations. This study used an untargeted metabolomics analysis by conducting ultra-high-performance liquid chromatography combined with quadrupole time-of-flight mass spectrometry (UHPLC-Q-TOF/MS) of sheep serum under overgrazing and light grazing conditions to identify metabolic disruptions in response to overgrazing.

**Results:**

The sheep body weight gains as well as serum biochemical variables associated with immune responses and nutritional metabolism (immunoglobulin G, albumin, glucose, and nonesterified fatty acids) were significantly decreased with overgrazing compared with light grazing condition. In contrast, other serum parameters such as alanine and aspartate aminotransferase, alkaline phosphatase, total bilirubin, blood urea nitrogen, and interleukin-8 were markedly higher in the overgrazing group. Principal component analysis discriminated the metabolomes of the light grazing from the overgrazing group. Multivariate and univariate analyses revealed changes in the serum concentrations of 15 metabolites (9 metabolites exhibited a marked increase, whereas 6 metabolites showed a significant decrease) in the overgrazing group. Major changes of fatty acid oxidation, bile acid biosynthesis, and purine and protein metabolism were observed.

**Conclusions:**

These findings offer metabolic evidence for putative biomarkers for overgrazing-induced changes in serum metabolism. Target-identification of these particular metabolites may potentially increase our knowledge of the molecular mechanisms of altered immune responses, nutritional metabolism, and reduced sheep growth performance under overgrazing conditions.

## Background

Overgrazing is the most severe threat to the typical steppe of Inner Mongolia. In recent decades, the grassland ecosystem as well as mutton and milk production have been extensively damaged due to overgrazing. Gains in the stocking rate have resulted in the accumulation of volatile organic compounds and morphological alterations in grassland plants [1]. Multiyear or single year research studies have revealed that overgrazing induces a significant decline in sheep (15-mo-old female) live body weight gain per hectare or individual in grazing season (June to September) [2, 3]. The reason for that is because overgrazing has severely damaged this natural grassland, which degrades the normal morphology of forages (lower forage production and imbalanced nutrients composition) resulting in reduction of the individual animal growth performance. With the growing emphasis on grazing animal welfare, increasing attention has been placed on the potential for the health status and nutrient metabolism of grazing sheep to be altered by increasd grazing intensity, especially under overgrazing conditions [4]. In response to overgrazing induced undernutrition, the long-term response of animal was characterized by nutrients mobilization (energy, protein and so on), which triggered tissue masses decrease, especially fat and muscle tissues. This may be a primary reason for the reduced growth performance observed in overgrazing sheep. Proteomics data obtained from our former study also demonstrated that overgrazing may trigger a shift in energy resources from carbohydrates to proteins due to the shortage supply of forage and imbalanced nutrients composition [4]. Furthermore, various proteins associated with immune response were down-regulated in the hepatic proteome of sheep in response to overgrazing [4]. A number of metabolites related to sheep immune responses and nutritional metabolism may be altered by overgrazing induced forage degradation and lower animal growth performance. However, few quantitative data are available on the metabolic profile alterations of sheep under overgrazing conditions.

Recently, metabolomics has become a powerful high-throughput bioanalytical method for detecting important metabolite biomarkers in ruminants in response to different diets or environmental stresses [5–7]. In a previous metabolomics study, analysis of serum and urine samples collected from sheep after 12 h and 48 h of road transport by nuclear magnetic resonance (NMR) spectroscopy revealed peroxisomal fatty acid oxidation as a metabolic response to transport-induced stress [8]. The metabolomic profile of rumen fluid in dairy cows fed a high-concentrate diet demonstrated altered concentrations of ruminal metabolites (bacterial degradation products, toxic compounds and amino acids) as well as an altered metabolic pattern [9]. Liquid chromatography/mass spectrometry (LC/MS) analysis of serum samples of lactating dairy cows under controlled heat stress revealed 13 potential biomarkers involved in carbohydrate, amino acid, lipid, or gut microbiome-derived metabolism, indicating that heat stress affected the metabolic pathways in lactating dairy cows [10]. To date, most metabolomics studies on ruminants focus on the zero-grazing system. However, this advanced bioanalytical method is still not widely used to evaluate alterations of the health status and nutritional metabolism of animals under overgrazing conditions.

Therefore, the goals of the present study were to identify and characterize the potential biomarkers in sheep serum that are associated with the consequences of overgrazing. The findings of this study provide critical information that may be utilized to improve the production of grazing animals and for the sustainable development of the Inner Mongolia steppe.

## Results

### Herbage nutritional contents, sheep growth performance and serum biochemical parameters

For herbage nutritional contents, crude protein (CP) and acid detergent lignin (ADL) were significantly higher in the overgrazing (OG) group than in the light grazing (LG) group (*P* < 0.05), nitrogen free extract (NFE) was significantly lower in the OG group than in the LG group (*P* < 0.05), and there was not significant difference in gross energy between the two groups (see Additional file 1). By the end of the grazing experiment, the body weight gain of sheep in the OG group was 12.2 kg, while that in the LG group was 14.9 kg (*P* < 0.05) (Table 1). For serum biochemical parameters, alanine aminotransferase (ALT), aspartate aminotransferase (AST), alkaline phosphatase (ALP), total bilirubin (TBIL), serum blood urea nitrogen (BUN) and interleukin-8 (IL-8) were significantly higher in the OG group than in the LG group (*P* < 0.05); albumin (ALB), glucose (GLU), nonesterified fatty acids (NEFAs) and immunoglobulin G (IgG) were significantly lower in the OG group than in the LG group (*P* < 0.05) (Table 2).

**Table 1:** Effect of overgrazing on the growth performance of sheep

Group	Body weight (kg)				Body weight gain (kg)	
	Initial	SEM	Final	SEM		SEM
LG	33.1 ± 1.1	0.3	48.1 ± 3.0^a^	0.9	14.9 ± 3.3^a^	0.9
OG	33.0 ± 4.7	1.4	45.4 ± 5.9^b^	1.7	12.2 ± 2.5^b^	0.7

Numbers are expressed as the means ± SDs. (*n* = 12). The means within a column with different superscript letters are significantly different at *P* < 0.05

**Table 2:** The effect of overgrazing on sheep serum biochemical variables

Item (serum activity/concentration)	LG	OG	SEM
ALT (IU/L)	28.51 ± 9.75^b^	39.30 ± 7.32^a^	2.27
AST (IU/L)	125.25 ± 13.03^b^	148.26 ± 15.30^a^	3.57
ALP (U/L)	203.43 ± 11.76^b^	270.71 ± 26.35^a^	3.89
ALB (g/L)	32.36 ± 4.78^a^	26.73 ± 4.21^b^	0.89
TBIL (mmol/L)	4.13 ± 0.66^b^	6.26 ± 1.37^a^	0.21
BUN (mmol/L)	8.75 ± 0.38^b^	11.20 ± 1.55^a^	0.26
GLU (mmol/L)	5.95 ± 1.26^a^	4.38 ± 0.87^b^	0.30
NEFAs (mmol/L)	0.62 ± 0.05^a^	0.37 ± 0.08^b^	0.02
IL-8 (pg/mL)	0.48 ± 0.07^b^	0.75 ± 0.11^a^	0.02
IgG (g/L)	17.12 ± 2.57^a^	13.79 ± 1.83^b^	0.45

*LG* light grazing, *OG* overgrazing

*ALT* alanine aminotransferase, *AST* aspartate aminotransferase, *ALP* alkaline phosphatase, *ALB* albumin, *TBIL* total bilirubin, *BUN* blood urea nitrogen, *GLU* glucose, *NEFAs* nonesterified fatty acid, *IL* interleukin, *IgG* immunoglobulin G

Numbers are expressed as the means ± SDs. (*n* = 12). The means within a row with different superscript letters differ significantly at *P* < 0.05

### Plasma metabolic profiling and different serum metabolites between the LG and OG groups

**Fig. 1:**
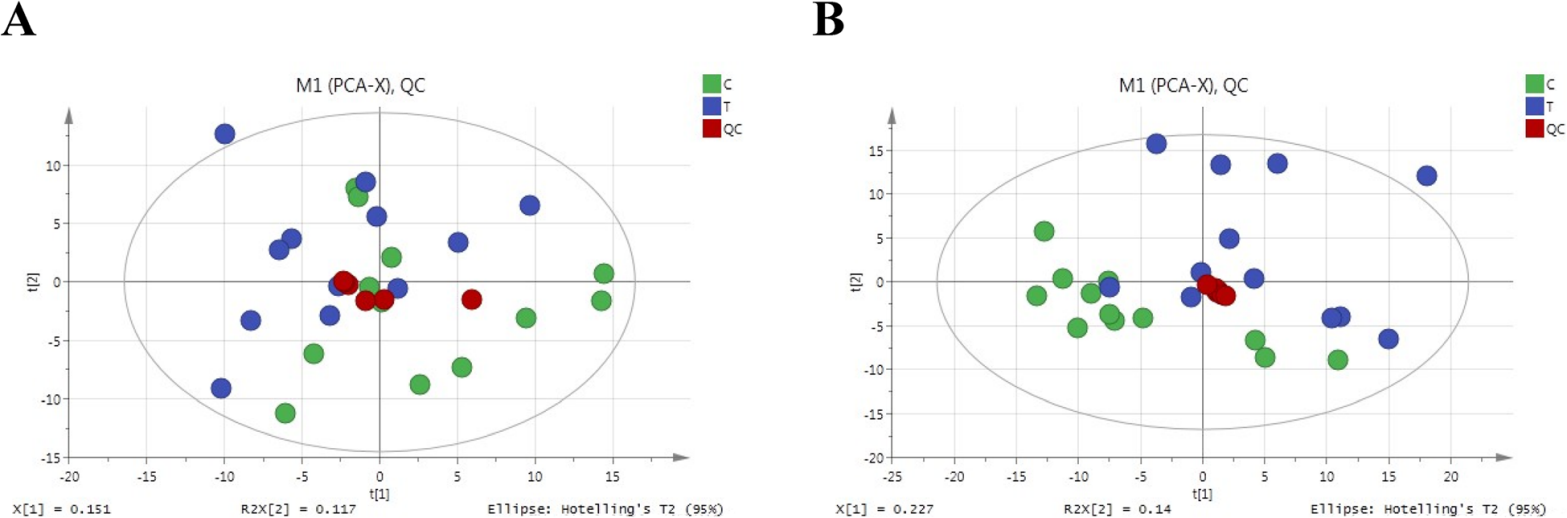
PCA scores in ESI positive mode (**a**) and ESI negative mode (**b**) based on the ultra-high-performance liquid chromatography combined with quadrupole time-of-flight mass spectrometry (UHPLC-Q-TOF/MS) data of the serum samples. Green dots: serum samples of sheep under LG conditions; blue dots: serum samples of sheep under OG conditions; red dots: QC samples

Figure 1 showed the distribution of metabolic profiles in the quality control (QC) samples from Pareto-scaled principal component analysis (PCA). Clear clustering of QCs in PCA space was shown for both the electrospray ionization (ESI) (+) and ESI (−) modes. The consistently repeated QC injections as well as reliable data quality among samples indicated the excellent repeatability, performance and stability of the sample treatment procedure.

**Fig. 2:**
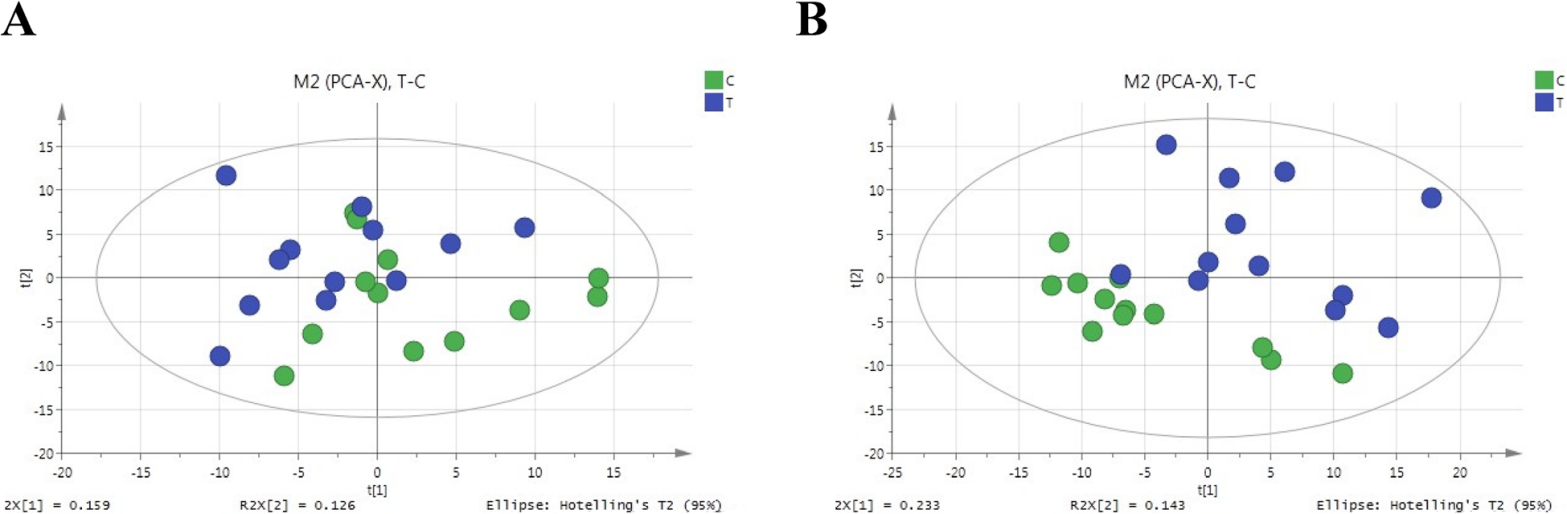
PCA score map derived from UHPLC-Q-TOF/MS spectra for LG sheep (green dots) and OG sheep (blue dots) in the positive mode (**a**) and negative mode (**b**)

To assess differences in global serum metabolite fingerprints between LG and OG sheep, the separation in both ion modes between the LG and OG sheep by the unsupervised PCA method was evaluated. As shown in Fig. 2, the two groups exhibited minimal but not significant differences in trend in the 2D PCA plots. We thus conducted further multivariate statistical analysis to establish the relationship between the two groups.

**Fig. 3:**
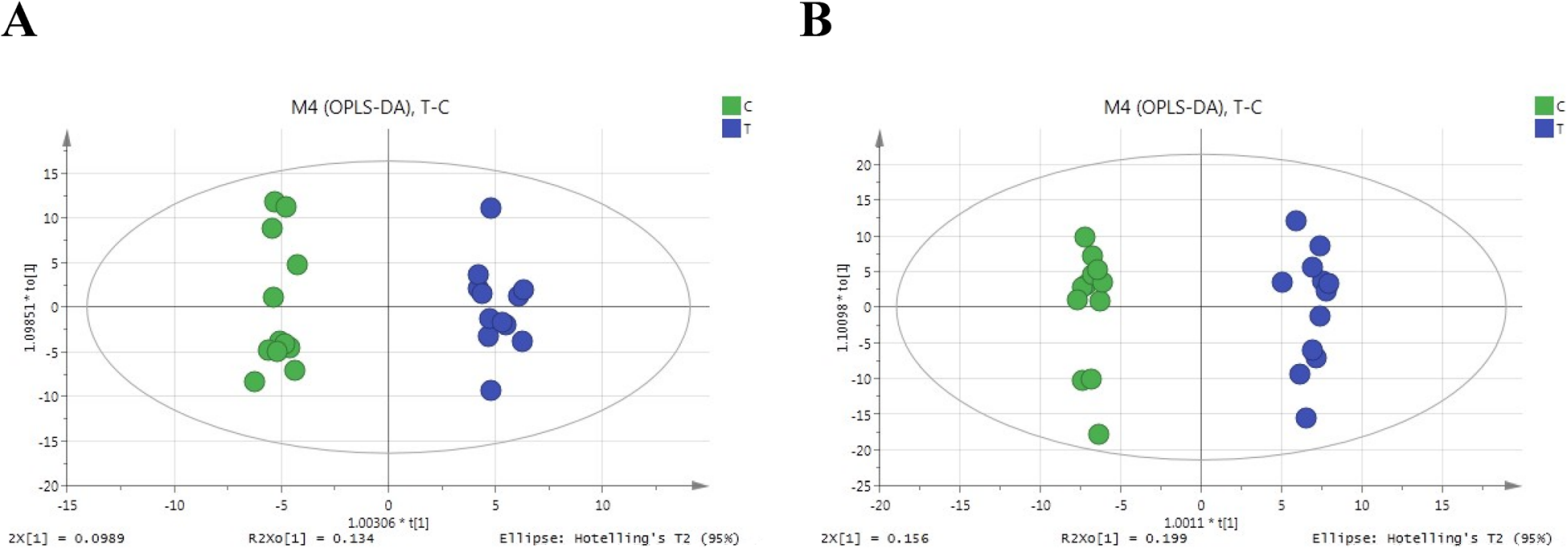
OPLS-DA score map derived from UHPLC-Q-TOF/MS spectra for LG sheep (green dots) and OG sheep (blue dots) in the positive mode (**a**) and negative mode (**b**)

Supervised orthogonal partial least-squares discriminant analysis (OPLS-DA) is a powerful method with which to discriminate ion peaks that contribute to the classification of samples and remove noncorrelated variations contained within spectra. Thus, OPLS-DA was conducted to confirm the separation and detect potential biomarkers between the LG and OG groups. Clear separation between the LG and OG groups based on the first two components was detected based on the OPLS-DA plot using the positive ion mode (R^2^Y = 0.984, Q^2^Y = 0.751) (Fig. 3a) and the negative ion mode (R^2^Y = 0.990, Q^2^Y = 0.869) (Fig. 3b), with R^2^Y indicating the goodness of the fit, with Q^2^Y indicating the model’s prediction ability. The models showed good sample classification into two groups, which was suggestive that the model was the high reliability and had predictive power. In both the PCA or OPLS-DA plot, the separation between the LG and OG groups was superior in both positive and negative modes, which indicated that sheep under OG conditions exhibited some alterations in the levels of a few metabolites.

Based on statistical analysis and the variable importance in the projection (VIP) value obtained from the OPLS-DA, 15 significantly different serum metabolites were selected as potential biomarkers related to OG (Table 3). Among the different alterations observed, the concentration of 6 metabolites was remarkably higher and of 9 metabolites was significantly lower in the OG group than in the LG group.

**Table 3:** List of significantly changed compounds in sheep from overgrazing group and light grazing group

Ionization mode	Metabolites	Adduct	VIP	Fold change	*P* value	Retention time (S)	m/z	Related pathway
ESI (+)	Stearoylcarnitine	(M + H)+	1.5381	1.8064	0.0347	254.1865	428.3754	Fatty acid beta-oxidation
ESI (+)	L-Palmitoylcarnitine	(M + H)+	1.4626	1.6676	0.0158	263.4055	400.3447	Fatty acid beta-oxidation
ESI (+)	Cholic acid	(M + NH_4_)+	10.7858	3.6272	0.0358	383.6785	426.3243	Primary bile acid biosynthesis
ESI (−)	Taurine	(M-H)-	1.1519	1.3343	0.0687	537.531	124.0072	Primary bile acid biosynthesis
ESI (+)	Adenosine	(M + H-H_2_O)+	1.2463	2.4131	0.0919	163.891	250.0973	Purine metabolism
ESI (−)	Hypoxanthine (HYPO)	(M-H)-	4.6457	0.4923	0.0350	304.1335	135.0318	Purine metabolism
ESI (−)	L-Tryptophan	(M-H)-	1.1472	0.8695	0.0608	469.231	203.0821	Biosynthesis of proteins
ESI (−)	D-Mannose	(M + CH_3_COO)-	1.3052	0.7540	0.0746	560.7445	239.0763	Antioxidation
ESI (−)	4-Pyridoxic acid	(M-H)-	3.0237	0.5923	0.0002	69.6135	182.0455	Vitamin B6 metabolism
ESI (+)	4-Pyridoxic acid	(M + H)+	2.9734	0.5609	0.0001	67.7175	184.0648	Vitamin B6 metabolism
ESI (+)	1-Stearoyl-sn-glycerol 3-Phosphocholine (LPC)	(M-H + 2Na)+	1.4861	1.4210	0.0037	283.888	568.3406	Major phospholipid components in serum (liver toxicity)
ESI (+)	1,2-Di-(9Z-octadecenoyl)-sn-glycero-3-phosphocholine (DOPC)	(M + H)+	11.7198	0.5475	0.0848	169.062	786.5999	Structure of cell membranes
ESI (+)	Acetylcarnitine	(M + H)+	3.1389	0.8104	0.0811	564.452	204.1271	Related to ammonia concentration
ESI (−)	Salicyluric acid	(M-H)-	2.9182	0.3989	0.0000	302.264	194.0461	Energy metabolism
ESI (−)	Salicylic acid	(M-H)-	7.7978	0.3045	0.0001	64.863	137.0247	Immune function modulation
ESI (−)	DL-Lactate	(M-H)-	11.3476	0.7210	0.0316	413.5235	89.02506	Central carbon metabolism in cancer

**Fig. 4:**
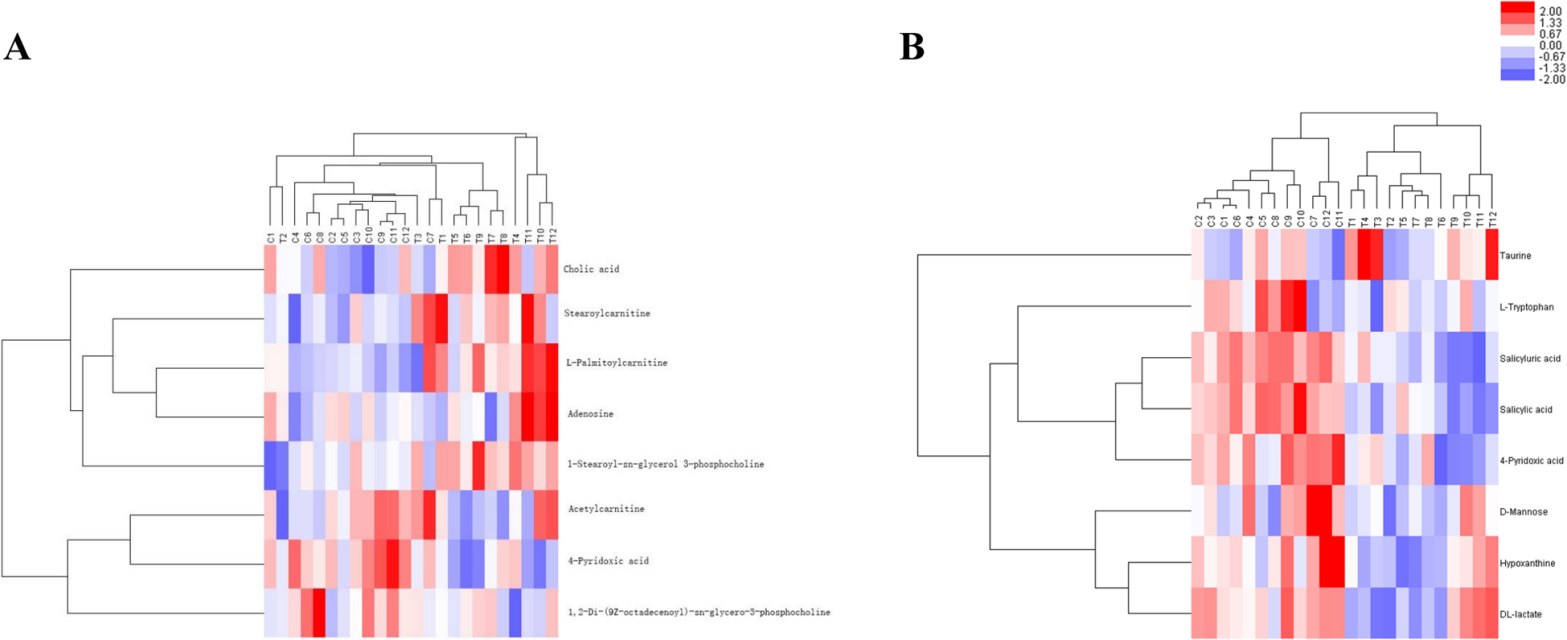
Unsupervised hierarchical clustering heat map of metabolites obtained from the serum of sheep in the positive mode (**a**) and negative mode (**b**)

To further evaluate alternations in the serum metabolome caused by OG, we conducted hierarchical clustering analysis using the degree of similarity in metabolite abundance profiles (Fig. 4a and b). Metabolites with identical abundance patterns were clustered together. The heatmap dendrogram revealed the tighter clustering of the OG-induced metabolites, including their separation from the LG group.

The identified metabolites influencing OG play a major role in particular metabolic pathways. Therefore, to determine possible pathways influenced by OG, the metabolites were assessed using the Kyoto Encyclopedia of Genes and Genomes (KEGG) pathway database (http://www.genome.jp/kegg/) (Table 3). Metabolomics analysis indicated that the variable effects of OG on various metabolic pathways in serum could be detected to follow an untargeted manner. These findings revealed that the potential biomarkers contribute to various processes, including fatty acid metabolism, bile acid synthesis, and purine and protein metabolism. (Table 3). Various metabolic pathways were thus induced by alterations in OG-induced sheep physiological function and might be closely associated with these changes.

## Discussion

The present study is to explore the biofluids of sheep under OG conditions to identify metabolic profiles that could be link to animal growth performance. Our previously published data demonstrated that various hepatic responses in sheep were triggered by OG (proteomic profiles) [4]. However, the serum metabolic changes of sheep under OG conditions were still largely unknown. In this study, LC/MS-based metabolomics was used to compare the metabolisms of sheep under LG and OG conditions, which will provide a comprehensive overview of the metabolic profile to help understand the molecular mechanisms of reduced animal growth performance in response to OG. Our results revealed that sheep growth performance as well as the serum metabolome was significantly influenced by OG. Furthermore, nutrients composition of pasture was also changed greatly under OG conditions, especially CP, ADL and NFE. The reason for this may be because the composition of plant community was altered by OG [11]. However, this speculation is still required to be demonstrated in future studies, which is not the focus of the present research.

Stearoylcarnitine and L-palmitoylcarnitine are long-chain acylcarnitines, which are markers of lipid oxidation that function as chaperones for fatty-acyl CoA transport into the mitochondria [12], can enhance energy and physical function as well as play a major role in the transfer and use of lipids [13]. Here, a higher level of stearoylcarnitine and L-palmitoylcarnitine was observed in the OG sheep than in the LG sheep, which reflected the elevated fatty acid metabolism in the former [14, 15]. This result was consistent with the lower serum levels of NEFAs in the present study and our previously obtained transcriptomic data (unpublished data). We found that several differentially expressed hepatic genes related to the peroxisome proliferator-activated receptor (PPAR) pathway and fatty acid oxidation were significantly up-regulated in the OG sheep. It was reported that additional energy utilized for animal physical activity would be necessary under increased grazing intensity, which would then reduce the available energy required for growth and production [16]. Therefore, the metabolomic and transcriptomic profiles described above may partially explain the reduced growth performance of sheep under OG conditions.

Bile acids (BAs) are a group of structurally similar compounds that function in lipid metabolism as well as intestinal signaling [17]. In addition, individual BA analysis may provide valuable information relating to the development of toxic liver injury and disease [18]. Cholic acid is one of the two major BAs produced by the liver [19]. The elevation of cholic acid is a potential biomarker of liver injury in various in vivo experimental models, and extra cholic acid may partially contribute to an increased risk of inflammation [20]. Taurine is involved in the primary BA biosynthesis pathway and conjugates with BAs to form bile salts, which are necessary for proper lipid absorption during digestion [21]. Higher levels of taurine have also been reported in several types of disease due to apoptosis [22–24]. Previous studies demonstrated that liver and gastrointestinal dysfunction disturbed hepatic synthesis and BA clearance, thereby resulting in alternations in serum BA profiles [25]. In the present study, classical biomarkers of hepatotoxicity, such as ALT, AST, ALP and TBIL, were significantly elevated in the OG group. The reason for this phenomenon may be the imbalanced nutritional contents of the herbage, especially higher CP and lower NFE contents in the herbage under OG conditions, which was also observed in our previous study [4]. Therefore, the increased levels of cholic acid and taurine suggest that OG may induce liver injury in sheep. However, it is reported that AST and ALT are not specific marker of liver damage in sheep [26]. Thus, direct evidence, such as hepatic tissue HE staining and immunohistochemical staining, is still needed, and will be performed in an additional study in the future.

Purine nucleosides participate in multiple intracellular neuronal processes and provide DNA and RNA precursors [27]. The primary role of purine metabolism is in the antioxidant defense system during neurodevelopment [28, 29]. Adenosine is a signaling nucleoside that is produced following tissue injury [30]. The production of extracellular adenosine and its subsequent signaling through adenosine receptors play an important role in orchestrating injury responses in a variety of organs [30]. Hypoxanthine is one of the end products in the catabolism of adenosine [31]. Lower levels of hypoxanthine can be considered a sign of less degradation of adenosine, indicating higher concentrations of adenosine in biofluids in the body [32]. In sleep loss or sleep deprivation experimental model groups, serum adenosine was elevated, but its product of degradation, hypoxanthine, was decreased, suggesting disordered nucleotide metabolism [33]. It was reported that with increasing grazing intensity, sheep decreased their time at rest to maintain their grazing time [16]. In the present study, an increase in the concentration of adenosine and a reduced level of hypoxanthine were observed in sheep serum under OG conditions, indicating that a lack of resting time due to OG might induce a disturbance of nucleotide metabolism in sheep.

A reduced level of L-tryptophan (Trp) was observed in the OG group in the current study. Trp is an important branched chain amino acid that is considered as a building block in protein synthesis as well as a vital biochemical precursor in multiple neurofunctional compounds [34]. It was recently reported that Trp was the third most limiting amino acid in growing lambs [35]. In a separate project, our laboratory detected differences in hepatic proteomics profiles between OG and LG groups [4]. Briefly, our observations demonstrated that a Trp catabolism enzyme (kynureninase, KYNU) [36] was more abundant in the hepatic proteome of OG sheep than in that of LG sheep. Thus, in the current study decreased levels of Trp under OG conditions reflect higher proteolysis or the impairment of protein synthesis, which is consistent with the lower concentration of serum TP in the OG group. Furthermore, lower levels of acetylcarnitine were observed in the OG group. Acetylcarnitine performs major functions as a cofactor for the transport of long-chain fatty acids through the mitochondrial membrane [37]. However, previous research has demonstrated that acetylcarnitine deficiency leads to increased ammonia levels in the body [38]. Our former research has also proved that the expression a hepatic protein (histidine ammonia-lyase, HAL) involved in ammonia elimination was increased in sheep under OG conditions [4]. Thus, above metabolomics and proteomics data could be a reason for the increased concentration of BUN in the OG group. Altered levels of stearoylcarnitine, L-palmitoylcarnitine, Trp and acetylcarnitine combined with the results of serum levels of NEFA, GLU and BUN indicate that sheep switch energy sources from carbohydrates to proteins and fatty acids under the OG condition, causing poorer nitrogen utilization efficiency and extra lipolysis. For energy metabolism, a number of studies revealed lower plasma GLU and ALB, and higher urea levels of ewes under restricted nutritional conditions [39–41]. This phenomenon was consistent with up-regulated expression of KYNU and HAL in our previously obtained hepatic proteomics data [4]. Thus, we speculate that Trp might be an important biomarker of OG, which could also explain the reduced sheep growth performance. In accordance with the Trp level, 4-pyridoxic acid and salicylic acid were reduced in OG sheep in the current study. Lower levels of 4-pyridoxic acid reflect vitamin B6 dysfunction [42]. The active form of vitamin B6 is involved in various enzyme reactions that are related to amino acid and lipid metabolism [43]. In contrast, defects in vitamin B6 affected antioxidant defenses in animal livers [44, 45]. Salicylic acid is a 5-aminosalycilic acid precursor and suppresses IL-8 secretion by TNF-α-activated intestinal epithelial cells [46]. In this study, increased concentration of IL-8 and reduction of 4-pyridoxic, salicylic acid and IgG were observed simultaneously in the OG group, which was consistent with the above research conclusion that sheep may be under environmental stresses due to the induction of certain inflammatory factors [47] and inadequate disease resistance under OG conditions. Previous research demonstrated that serum IgG concentration had significant linear association with buffalo calves average daily gain (ADG) from birth to day 30 [48]. Therefore, the lower levels of 4-pyridoxic, salicylic acid and IgG integrated with higher level of IL-8 in the present study may be induced by the reduction of sheep growth performance under OG conditions. Our previous hepatic proteomics data showed that the expression of proteins involved in immune and inflammatory responses were downregulated and upregulated, respectively, in the OG sheep [4], which was consistent with the metabolomic data obtained in the present study. However, it is important to note that this study reported immune and inflammatory responses in whole blood that does not entirely indicate functional changes. Thus, these results should be interpreted with caution and further research study is needed to confirm the effect of OG on innate and adaptive immunity using specific immune cells such as neutrophils, rather than whole blood [47].

## Conclusions

In conclusion, sheep under the OG condition exhibited significantly reduced growth performance and evident changes of serum biochemical variables compared to sheep under the LG condition. Simultaneous changes of serum metabolites have also been identified by untargeted metabolomics using UHPLC-Q-TOF/MS, thereby providing a unique perspective concerning OG-triggered changes in cellular metabolites. Changes in 15 serum metabolites (6 metabolites exhibited a significant increase in concentration, and 9 metabolites showed a significant reduction in concentration) were identified in sheep under the OG condition and were mainly involved in fatty acid oxidation, bile acid biosynthesis and purine and protein metabolism. The identification of the targets of these metabolites improves our understanding of the molecular mechanism of reduced sheep growth performance under OG conditions.

## Methods

### Animals and experimental design

The present study was conducted following the Regulations for the Administration of Affairs Concerning Experimental Animals of the State Council of the People’s Republic of China. The Committee on Experimental Animal Management of the Chinese Academy of Agricultural Sciences, (Beijing), approved our research protocol (Approval No. 46/28.05.2016).

We conducted this research at the Institute of Grassland Research of the Chinese Academy of Agricultural Sciences Research Station in 2016 during the summer. The site is located at the Xilin River basin in the Inner Mongolia Autonomous Region of China (116°32′ E, 44°15′ N). Its predominant natural vegetation included three species of grass: *Leymus chinensis*, *Stipa krylovii*, and *S. grandis*.

**Fig. 5:**
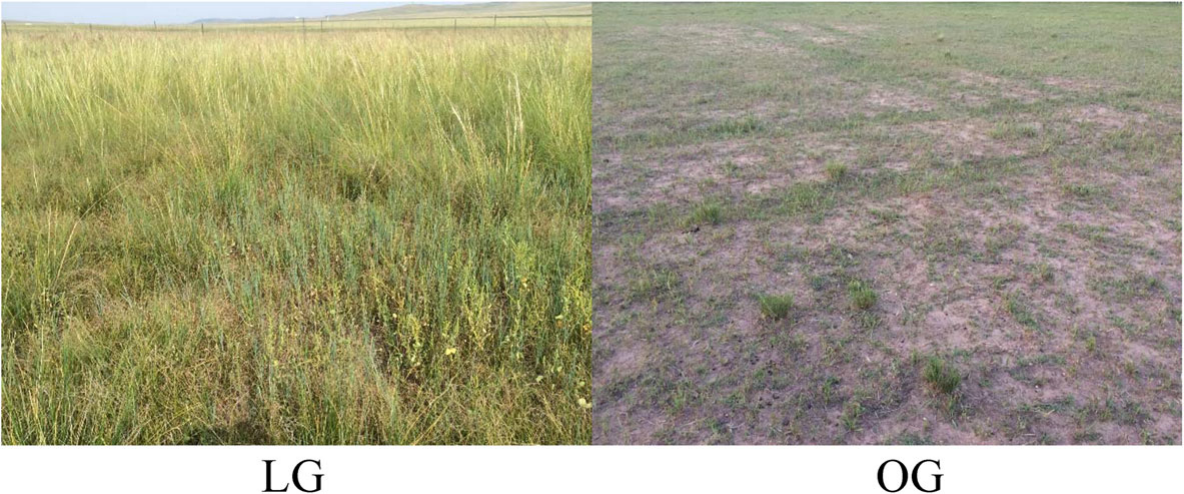
Grazing differences between LG plots and OG plots

In June 2016, a total of 48 Uzhumchin wethers (average age: 24 months; baseline live weight (LW):33.2 ± 3.9 kg) were obtained from the Xinshengjia Sheep Breeding Company (Xilinhot, Inner Mongolia, China). We randomly assigned the wethers to either the LG (4 sheep/plot) or the OG (12 sheep/plot) groups. The experimental site comprised six plots (1.33 ha/plot, three plots per grazing group). Two different stocking rates were used, namely, 3.0 (LG) and 9.0 sheep/ha (OG) (Fig. 5). The wethers were allowed to graze without interruption in the plots during the duration of the experiment (June 5 to September 3, 90 days). At the start of the research study, sheep were treated with an anthelmintic to eliminate the infection of internal parasites [2] and given water and mineral lick stones ad libitum. After the grazing experiment, all sheep were released.

### Sample collection

To identify the chemical constituents of the sheep diet, we collected plants every month of the grazing experiment. The protocol for plant collection was as described elsewhere [49] and the details are provided as supplementary material (see Additional file 2). The plants were assessed for different chemical composition indices such as CP, gross energy, NFE, neutral detergent fiber (NDF), acid detergent fiber (ADF), as well as ADL, as described elsewhere (a single pooled sample per each plot, *n* = 3) [2]. The LW of the animals was identified on the study’s first and the last day (after 12 h of fasting) and used it to calculate the mean daily gain. Blood was then collected from four sheep selected at random from each plot in the groups (*n* = 12 for each group) via venipuncture of the jugular vein and into vacuum tubes. The blood was then centrifuged twice at *2000 g* at 4 °C for 30 min and then at *400 g* at 4 °C for 10 min to isolate the sera, which were then kept at − 80 °C to await further analysis.

### Biochemical, immune response and inflammation analyses

For biochemical, immune response and inflammatory variables of the serum, the concentration of ALT, AST, ALP, ALB, TBIL, BUN, GLU, and NEFA were measured using a fully automatic biochemistry analyzer (Hitachi 7020, Tokyo, Japan); IL-8 and IgG were determined using a corresponding diagnostic kit (Nanjing Jiancheng Bioengineering Institute, Nanjing, China) according to the instructions of the manufacturer.

### Sample preparation

Each sample was slowly thawed at 4 °C. One volume of plasma (50 μL) was mixed with three volumes of cold mixtures of methanol/acetonitrile/H_2_O (2:2:1, v/v/v) to precipitate proteins following adequate vortexing, incubation (− 20 °C for 1 h) and centrifugation (15 min at 13,000 rpm and 4 °C). The supernatant was collected, dried in a vacuum, redissolved in 50 μL of acetonitrile/water (1:1, v/v), and centrifuged at 14,000 rpm for 15 min, 4 °C. A pooled QC sample was created by taking the same amount of each sample and mixing the amounts together. We employed QC samples to evaluate the system’s stability and performance before sample loading and during the whole experimental process (every five samples).

### Metabolomics analysis

We conducted sample analyses using an ultra-high-performance liquid chromatography (UHPLC) system (Agilent 1290 Infinity LC system, USA) coupled with a premium quadrupole time-of-flight mass spectrometer (QTOF MS) system (AB Sciex TripleTOF 6600, MA, USA). Hydrophilic interaction liquid chromatography (HILIC) separation of metabolite samples was performed on a reversed-phase C_18_ ACQUITY UPLC BEH column (2.1 mm × 100 mm, 1.7 μm; Waters, Ireland). In both the ESI positive and negative modes, the flow rate was 400 μL/min, with solvent A composed of 25 mM ammonium acetate + 25 mM ammonium hydroxide and solvent B composed of acetonitrile. The gradient consisted of 85% B for 1 min, a linear reduction to 65% B in 11 min, a reduction to 40% B in 0.1 min, holding this concentration for 4 min, and then an increase to 85% B in 0.1 min, with a 5 min re-equilibration period.

MS was operated with an AB Sciex TripleTOF mass spectrometer equipped with heated ESI positive and negative modes (AB Sciex TripleTOF 6600). The conditions of the ESI source were the following: ion source gas 1 (gas 1), 60; ion source gas 2 (gas 2), 60; curtain gas (CUR), 30; source temperature, 600 °C, and ion spray voltage floating (ISVF), ± 5500 V. For MS-only acquisition, the instrument used an m/z range of 60–1000 Da, with an accumulation time for the TOF MS scan was set at 0.20 s/spectra. For auto MS/MS acquisition, the instrument was set to within an m/z range of 25–1000 Da, with an accumulation time of 0.05 s/spectra. We conducted the product ion scan with information-dependent acquisition (IDA) at a mode of high sensitivity. Collision energy (CE) was set at 35 V and ± 15 eV. The declustering potential (DP) was at ±60 V.

### Data processing and analysis

The raw MS data using an instrument-specific format (.wiff) were converted to the common data format (MzXML) with the ProteoWizard tool msconvert (version 3.0.10051) and then processed by the program XCMS for feature assessment, retention time correction, as well as alignment. Metabolite identification was performed based on the accurate mass (< 25 ppm) and MS/MS data that were matched to our standard database. In ion features that were extracted, the subsequent analysis included only the variables showing > 50% of the nonzero measurement values in one or more groups.

After normalization that used total peak intensity, we uploaded the processed data to SIMCA-P (version 14.1, Umetrics, Umea, Sweden) for multivariate data analysis, including PCA as well as OPLS-DA. Seven-fold cross-validation as well as response permutation testing were employed to assess model robustness. The VIP value of every variable in the OPLS-DA model was assessed to determine its the contribution to classification. Furthermore, metabolites with a VIP value > 1 were assessed using the Student’s *t*-test at the univariate level to determine their significance [the normality test of data was conducted using SPSS Statistics 17.0 (SPSS, Inc., USA)], and a *P* value < 0.05 was deemed as statistically significant.

## Supplementary information

**Additional file 1: Table S1.** Effect of overgrazing on primary nutritional indexes of herbage.

**Additional file 2.** The detailed description of forage sample collection.

## Data Availability

The datasets used and/or analyzed during the current study are available from corresponding author on reasonable request.

## Abbreviations

KEGG: Kyoto encyclopedia of genes and genomes
LG: Light grazing
OG: Overgrazing
OPLS-DA: Orthogonal partial least-squares discriminant analysis
PCA: Principal component analysis
