# Supplementary material for: Comparative metabolome analysis of serum changes in sheep under overgrazing or light grazing conditions
Source: BMC Vet Res. 2019 Dec 26;15:469. doi: 10.1186/s12917-019-2218-9 (PMC6933664; doi:10.1186/s12917-019-2218-9)
Supplement: Supplementary file 1 — Additional file 1: Table S1. Effect of overgrazing on primary nutritional indexes of herbage. [file 12917_2019_2218_MOESM1_ESM.doc]

**Tables S1 Effect of overgrazing on primary nutritional indexes of herbage**

|  | Groups | |  |
| --- | --- | --- | --- |
|  | LG | OG | SEM |
| CP (g/kg DM) | 85.3 ± 7.2^b^ | 121.2 ± 8.9^a^ | 4.7 |
| Gross energy (kJ/g DM) | 18.06 ± 0.17 | 17.43 ± 0.52 | 0.22 |
| NFE (g/kg DM) | 47.8 ± 3.9^a^ | 41.5 ± 2.2^b^ | 1.8 |
| NDF (g/kg DM) | 608.7 ± 32.5 | 552.9 ± 45.6 | 22.9 |
| ADF (g/kg DM) | 285.3 ± 17.2 | 319.5 ± 16.0 | 9.8 |
| ADL (g/kg DM) | 245.5 ± 24.7^b^ | 308.4 ± 15.9^a^ | 11.9 |

LG, light grazing; OG, overgrazing; CP, crude protein; NFE, nitrogen free extract; NDF, neutral detergent fibre; ADF, acid detergent fibre; ADL, acid detergent lignin; Values within a row not sharing a common superscript letter indicate significant difference at *P* < 0.05. Numbers are means ± SD. (n = 3).
